# Intermuscular adipose tissue accumulation is associated with higher tissue sodium in healthy individuals

**DOI:** 10.14814/phy2.16127

**Published:** 2024-07-03

**Authors:** Lale A. Ertuglu, Melis Sahinoz, Aseel Alsouqi, Serpil Muge Deger, Andrew Guide, Mindy Pike, Cassianne Robinson‐Cohen, Elvis Akwo, Michael Pridmore, Rachelle Crescenzi, Meena S. Madhur, Annet Kirabo, David G. Harrison, Friedrich C. Luft, Jens Titze, T. Alp Ikizler, Jorge L. Gamboa

**Affiliations:** ^1^ Department of Medicine Vanderbilt University Medical Center Nashville Tennessee USA; ^2^ Now with Division of Hematology and Oncology, Department of Medicine University of Pittsburgh Medical Center Pittsburgh Pennsylvania USA; ^3^ Division of Nephrology, Department of Medicine Dokuz Eylul University Izmir Turkey; ^4^ Department of Biostatistics Vanderbilt University Medical Center Nashville Tennessee USA; ^5^ Division of Epidemiology, Department of Medicine Vanderbilt University Nashville Tennessee USA; ^6^ Division of Nephrology and Hypertension, Department of Medicine Vanderbilt University Medical Center Nashville Tennessee USA; ^7^ Department of Radiology and Radiological Sciences Vanderbilt University Medical Center Nashville Tennessee USA; ^8^ Department of Biomedical Engineering Vanderbilt University Nashville Tennessee USA; ^9^ Division of Clinical Pharmacology, Department of Medicine Vanderbilt University Medical Center Nashville Tennessee USA; ^10^ Department of Molecular Physiology and Biophysics Vanderbilt University Medical Center Nashville Tennessee USA; ^11^ Experimental and Clinical Research Center MDC/Charité Berlin Germany; ^12^ Program in Cardiovascular and Metabolic Disorders Duke NUS Medical School Bukit Merah Singapore

**Keywords:** inflammation, intermuscular adipose tissue, tissue sodium accumulation

## Abstract

**Background and Aims:**

High tissue sodium accumulation and intermuscular adipose tissue (IMAT) are associated with aging, type 2 diabetes, and chronic kidney disease. In this study, we aim to investigate whether high lower‐extremity tissue sodium accumulation relates to IMAT quantity and whether systemic inflammatory mediators and adipocytokines contribute to such association.

**Methods:**

Tissue sodium content and IMAT accumulation (percentage of IMAT area to muscle area) were measured in 83 healthy individuals using sodium imaging (^23^Na‐MRI) and proton (1H‐MRI) imaging of the calf. Insulin sensitivity was assessed by glucose disposal rate (GDR) measured with the hyperinsulinemic‐euglycemic clamp.

**Results:**

Median (interquartile range) muscle and skin sodium contents were 16.6 (14.9, 19.0) and 12.6 (10.9, 16.7) mmol/L, respectively. Median IMAT was 3.69 (2.80, 5.37) %. In models adjusted for age, sex, BMI, GDR, adiponectin, and high‐sensitivity C‐reactive protein, increasing tissue sodium content was significantly associated with higher IMAT quantity (*p* = 0.018 and 0.032 for muscle and skin tissue sodium, respectively). In subgroup analysis stratified by sex, skin sodium was significantly associated with IMAT only among men. In interaction analysis, the association between skin sodium and IMAT was greater with increasing levels of high‐sensitivity C‐reactive protein and interleukin‐6 (*p* for interaction = 0.022 and 0.006, respectively).

**Conclusions:**

Leg muscle and skin sodium are associated with IMAT quantity among healthy individuals. The relationship between skin sodium and IMAT may be mediated by systemic inflammation.

## Graphical abstract

Intermuscular adipose tissue and tissue sodium in healthy individuals.

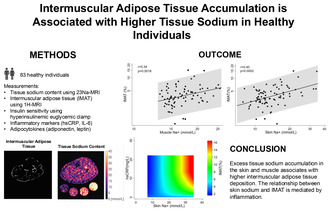

## 1 INTRODUCTION

Intermuscular adipose tissue (IMAT), an ectopic fat deposition beneath the deep fascia of the muscle, is observed in various conditions including aging (Song et al., 2004), obesity, insulin resistance (Therkelsen et al., 2013), chronic kidney disease (Gamboa et al., 2020), and type 2 diabetes (Bittel et al., 2015; Hilton et al., 2008). IMAT, like visceral adipose tissue, is associated with inflammatory cell infiltration and the secretion of pro‐inflammatory cytokines (Kelley & Goodpaster, 2015; Sachs et al., 2019). The severity of IMAT is also a predictor of muscle dysfunction and immobility in these chronic conditions (Addison et al., 2014). The mechanism underlying IMAT accumulation in these diverse conditions remains elusive, hindering the search for a therapeutic option to slow its progression.

Recent advances in our understanding of the body sodium handling suggest that sodium can accumulate in the tissue without commensurate water retention, particularly in the muscle and skin, which can be measured and quantified using ^23^Na MRI. Increased sodium deposition in the skin and muscle is associated with aging, type 2 diabetes, and chronic kidney disease (Kopp et al., 2013, 2018; Sahinoz et al., 2020), conditions that are also associated with increased IMAT (Cheema et al., 2010; Gamboa et al., 2020; Sachs et al., 2019). High sodium concentrations in vitro lead to the upregulation of adipogenesis and expression of inflammatory cytokines by adipocytes (Lee et al., 2019). Furthermore, a high concentration of extracellular sodium is a driver of immune cell activation and inflammation (Barbaro et al., 2017), which is also closely related to adipose tissue dysregulation (Kawai et al., 2021). In clinical studies, high salt intake has a well‐established association with increased visceral and subcutaneous adiposity through unclear mechanisms (Li et al., 2022; Ma et al., 2015; Zhu et al., 2014).

Given the similar disease states leading to tissue sodium and fat deposition, we hypothesized that excess tissue sodium accumulation is associated with increased fat deposition in the muscle. To test this hypothesis, we examined the tissue sodium content in the muscle and skin, assessed by ^23^Na MRI, and IMAT quantity in healthy individuals. We further investigated the potential influences of sex, serum inflammatory markers, and adipokines on these relationships.

## 2 METHODS

### 2.1 Study design and population

This was a post hoc analysis of a randomized clinical trial conducted at Vanderbilt University Medical Center (VUMC) from September 2014 to May 2018 (NCT02236520) (Alsouqi et al., 2022). A cross‐sectional investigation was carried out on 83 subjects who completed the study. All participants were between the ages of 30 and 80 years. Obesity was defined as body mass index (BMI) ≥30 kg/m^2^ established in the evidence report of the National Institutes of Health on the identification, evaluation, and treatment of overweight and obese adults as well as in the American College of Cardiology/American Heart Association Task Force on Practice Guidelines (Clinical Guidelines on the Identification, 1998; Jensen et al., 2014). Exclusion criteria included impaired kidney function (glomerular filtration rate <60 mL/min), impaired liver function, diabetes mellitus, acute cardiovascular events within the last 6 months, systemic glucocorticoid therapy within the last month, BMI less than 18 or greater than 40 kg/m^2^, contraindications to undergo magnetic resonance imaging (MRI), and use of antihypertensive therapy or insulin‐sensitizing medications. Written informed consent was obtained from all participants and the study was approved by the institutional review board at VUMC.

### 2.2 Hyperinsulinemic euglycemic clamp

Hyperinsulinemic‐euglycemic clamp technique, adapted from DeFronzo et al. (1979), was used to measure insulin sensitivity. The detailed methodology of the clamp has been published (Deger et al., 2018).

All study procedures were conducted at the Clinical Research Center at VUMC following an overnight hour fasting period. On the morning of the study day, peripheral intravenous access was obtained for the infusion of insulin and dextrose. After baseline blood samples were obtained, a primed infusion of regular human insulin at the concentration of 2.0 mU/kg/min of body weight was started and continued throughout the study to maintain hyperinsulinemia with a goal plasma insulin of 100 μU/mL. Plasma glucose concentration was monitored every 5 min. Dextrose 20% in water infusion was adjusted to reach and maintain the target plasma glucose levels of 90 ± 5 mg/dL. Once steady state was reached, the dextrose infusion rate was held constant for 30 min and insulin‐mediated glucose disposal rate (GDR) (mg/kg/min) was calculated from samples taken during this period. The average time to reach the steady state was 111 min. GDR was normalized to body weight and used as the index of insulin sensitivity.

### 2.3 Blood samples

All blood sampling was performed at the Clinical Research Center and analyzed at VUMC central laboratories. Leptin samples were analyzed at Vanderbilt's Hormonal Lab Core. The lipid profiles were obtained by using the Bruker IVDr Lipoprotein Subclass Analysis (B.I.LISA) method (Aru et al., 2017). High‐molecular‐weight adiponectin and interleukin 6 (IL‐6) were measured by enzyme‐linked immunosorbent assay (R&D Systems, Minneapolis, MN; catalog number DHWAD0 and D6050, respectively). High‐sensitivity C‐reactive protein (hsCRP) was measured by high‐sensitivity particle‐enhanced turbidimetric UniCel DxI Immunoassay system (Beckman Coulter) at the Vanderbilt Clinical Laboratory.

### 2.4 Magnetic resonance imaging

All participants underwent an MRI exam on a 3.0T scanner (Philips Healthcare, Best, The Netherlands). The exam consisted of multi‐nuclear sodium imaging (^23^Na‐MRI) using a single‐tuned receive‐only quadrature sodium coil (Rapid Biomedical GmbH, Rimpar, Germany), and proton (1H‐MRI) imaging of the calf. Participants were positioned supine to image the calf at the mid‐gastrocnemius muscle. Image analysis was accomplished through manual segmentation of the muscle and skin areas on the water‐weighted Dixon MRI image, which was acquired in the same field‐of‐view as the sodium MRI protocol. Tissue sodium content was calculated from standardized calibration of the ^23^Na‐MRI signal intensity to saline standards (10, 20, 30, and 40 mmol/L), and manual regional analysis in the skin and muscle (Crescenzi et al., 2020). The manual segmentations of skin and muscle were then applied as binary masks to the calculated tissue sodium content map to calculate tissue sodium content in the muscle and skin areas, respectively. IMAT was segmented using an automated unsupervised learning algorithm, specifically k‐means clustering, to separate areas of IMAT from the muscle segmentation in the fat‐weighted Dixon MRI image. The IMAT segmentation was then used to calculate the percentage of IMAT area relative to total muscle area (%) at the level of the mid‐calf (Gamboa et al., 2022; Sahinoz et al., 2020).

### 2.5 Dietary recall

The Nutrition Data System for Research software version 2017 (Nutrition Coordinating Center, University of Minnesota, Minneapolis, MN) was used for the collection and analysis of data on dietary intake (Schakel et al., 1988). 7‐day averages were calculated using two 24‐h dietary recalls obtained in‐person and over the telephone (one weekday and one weekend).

### 2.6 Statistical analysis

Participant characteristics were described as median (interquartile range) or number (percentage). Spearman's rank correlation was used to evaluate the correlation between tissue sodium content and IMAT quantity. Linear regression analysis was performed to estimate simple associations between IMAT quantity and tissue sodium content on a log scale to address non‐normal distribution. Scatter diagrams of IMAT and tissue sodium and line of best fit (with 95% confidence intervals) were plotted. In multivariable models, log‐transformed IMAT quantity was used as the primary dependent variable. The main independent variables were log‐transformed tissue sodium content modeled linearly. Adjusted models included demographics (age and sex), BMI (weight [kg]/height [m] (Therkelsen et al., 2013)), log‐transformed GDR, log‐transformed adipocytokines (adiponectin and leptin), and log‐transformed inflammatory markers (hsCRP and IL‐6).

To investigate the potential effect modification of the association of muscle and skin sodium content with IMAT quantity by inflammatory markers, linear models were fitted with the following interaction terms: skin/muscle sodium*hsCRP or skin/muscle sodium*IL‐6. The same analysis was repeated using sex in place of inflammatory markers. Sub‐group analysis was conducted stratified by sex and obesity.

Complete case analyses were performed for missing data. There were six missing values for hsCRP, two for adiponectin, and one for IL‐6. Dietary sodium data were missing in four participants. Analyses were performed using Stata version 16.0 (StataCorp LLC, College Station, TX). The level of significance was set as *p* < 0.05 (two‐sided).

## 3 RESULTS

### 3.1 Baseline characteristics

Eighty‐three participants were included in the final analysis (Table 1). Of these, 67.5% were female and 41% were African American. 87% of the female participants were premenopausal. Median (interquartile range [IQR]) BMI was 27.5 (24.4, 32.3) kg/m^2^ and 30 of the participants were obese. Median (IQR) muscle and skin sodium content measured by ^23^Na‐MRI were 16.6 (14.9, 19.0) and 12.6 (10.9, 16.7) mmol/L, respectively. Median (IQR) IMAT quantity was 3.69 (2.80, 5.37) %. Men were found to have higher mean IMAT as well as skin sodium content compared to women, as reported previously (Kopp et al., 2013; Machann et al., 2005). No difference was observed in muscle sodium content between men and women. There was no racial difference in IMAT accumulation or tissue sodium content.

**TABLE 1:** Baseline characteristics of study participants.

Characteristic	Overall (*n* = 83)	Female (*n* = 56)	Male (*n* = 27)
Age (years)	48 (36,58)	44.5 (34, 54.5)	52 (38, 62)
Postmenopausal (%)	–	10 (12.9)	–
African American origin (%)	34 (41)	25 (44.6)	34 (64.2)
BMI (kg/m^2^)	27.5 (24.4, 32.3)	27.5 (25, 31.5)	27.4 (24.1, 32.5)
SBP (mmHg)	126 (120, 132)	124 (119, 130.5)	124 (118, 129)
DBP (mmHg)	77 (71, 82)	76.5 (70, 81)	74 (70, 81)
Muscle Na+ (mmol/L)	16.6 (14.9, 19.0)	16.8 (14.7, 19.0)	16.3 (15.5, 18.9)
Skin Na+ (mmol/L)	12.6 (10.9,16.7)	12 (10.7, 13.8)	17 (14.9, 19.9)
IMAT (%)	3.69 (2.80, 5.37)	3.40 (2.67, 4.86)	4.38 (3.41, 6.33)
Leptin (ng/mL)	27.9 (15.5, 52.4)	36.8 (22.9, 64.0)	16.7 (8.9, 23.9)
Adiponectin (μg/mL)	18.6 (9.3, 35.5)	24.2 (12.2, 44.9)	11.1 (7.3, 23.4)
hsCRP (mg/dL)	1.3 (0.6, 3)	1.3 (0.5, 3.6)	1.6 (0.7, 2.4)
IL‐6 (pg/mL)	1.7 (1.0, 2.6)	1.9 (1, 3.1)	1.7 (1.2, 2.3)
Triglycerides (mg/dL)	74.7 (57.9, 116.5)	70.6 (54.2, 103.2)	89.0 (61.6, 146.9)
Total cholesterol (mg/dL)	188.3 (163.7, 216.7)	182.1 (165.4, 210.7)	193.8 (163.3, 217.1)
LDL (mg/dL)	108.5 (91.0, 129.6)	107.5 (85.5, 124.3)	108.5 (95.3, 140.9)
HDL (mg/dL)	55.3 (48.0, 63.9)	58.3 (50.0, 67.4)	51.3 (42.4, 55.3)
Creatinine (mg/dL)	0.83 (0.75, 0.91)	0.79 (0.71, 0.85)	0.90 (0.86, 0.99)
eGFR (mL/min/1.73 m)	97.2 (88.1, 107.0)	97.31 (88.31, 106.19)	95.1 (88.0, 109.5)
GDR (mg/kg/min)	8.44 (5.95, 11.47)	8.74 (6.21, 11.53)	6.85 (4.73, 11.19)
Dietary Na+ (mg/day)	2828 (2183, 3813)	2768 (2157, 3706)	2856 (2292, 3917)

Abbreviations: BMI, body mass index; DBP, diastolic blood pressure; eGFR, estimated glomerular filtration rate; GDR, glucose disposal rate; HDL, high‐density lipoprotein; hsCRP, high sensitivity C‐reactive protein; IL‐6, interleukin‐6; IMAT, intermuscular adipose tissue; LDL, low‐density lipoprotein; SBP, systolic blood pressure.

### 3.2 Correlation of dietary sodium intake with tissue sodium and IMAT

The median (IQR) dietary sodium intake was 2827 (2183–3813) mg/day. Dietary sodium intake did not correlate with muscle or skin sodium content (*r* = 0.14, *p* = 0.2 and *r* = 0.07, *p* = 0.6 for muscle and skin, respectively). No correlation was found between IMAT quantity and dietary sodium intake (*r* = 0.15, *p* = 0.19).

### 3.3 Correlations of IMAT with metabolic risk factors

There was a significant positive correlation between IMAT quantity and BMI (*r* = 0.30, *p* = 0.005). No correlation was found between IMAT and plasma triglyceride, total cholesterol, LDL or HDL cholesterol. IMAT did not correlate with plasma adiponectin level (*r* = −0.19, *p* = 0.09) or leptin levels (*r* = −0.05, *p* = 0.66). No correlation was found between IMAT and serum inflammatory markers hsCRP and IL‐6. There was a significant negative correlation between IMAT and GDR (*r* = −0.34, *p* = 0.002).

### 3.4 Relationship of tissue sodium content with IMAT

**FIGURE 1:**
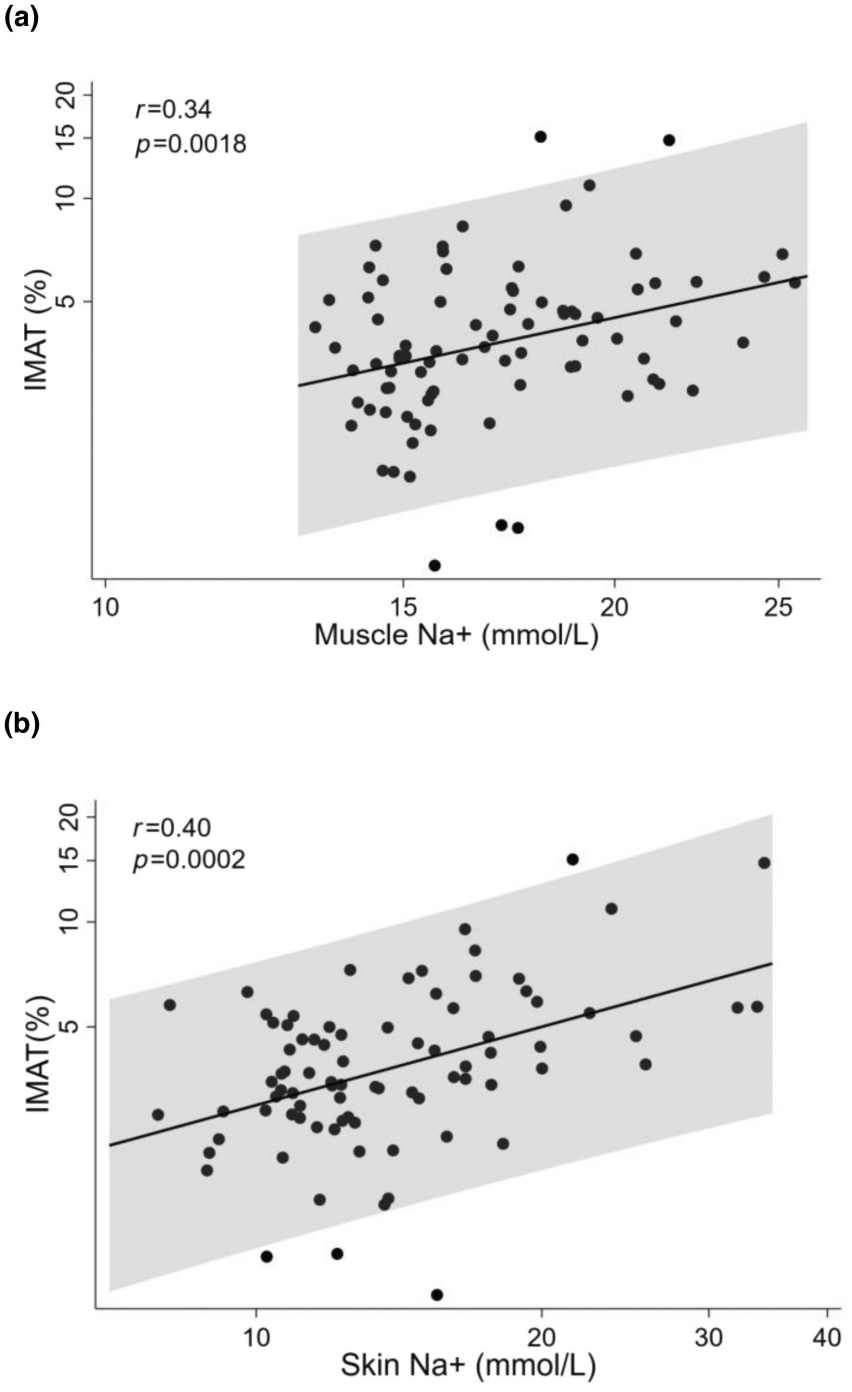
The regression plots of muscle (a) and skin (b) sodium with IMAT. Plots presented with linear regression line along with 95% confidence intervals. IMAT positively correlated with muscle and skin sodium (*r* = 0.34, *p* = 0.0018 and *r* = 0.40, *p* = 0.0002, respectively).

IMAT quantity was positively correlated with muscle and skin sodium (*r* = 0.34, *p* = 0.0018 and *r* = 0.40, *p* = 0.0002, respectively; Figure 1). A 10% increase in muscle sodium corresponded to a 10.7% increase in IMAT in univariate regression analysis. Similarly, a 10% increase in skin sodium content corresponded to a 7.4% increase in IMAT. In multivariable linear regression models adjusted for age, sex, BMI, GDR, adiponectin, and hsCRP, the associations between skin and muscle sodium, and IMAT remained statistically significant (Table 2). The associations remained significant in the second model adjusted for age, sex, BMI, GDR, leptin, and IL‐6. Skin or muscle sodium did not correlate with systemic markers of adiposity including BMI, leptin, or adiponectin.

**TABLE 2:** The associations between IMAT quantity and tissue sodium content.

IMAT%				
	Muscle Na+		Skin Na+	
	β (95% CI)	*p*‐value	Β (95% CI)	*p*‐value
Unadjusted				
Tissue Na+	1.06 (0.38, 1.75)	0.003	0.75 (0.42, 1.08)	0.000
Adjusted				
Tissue Na+	0.83 (0.15, 1.51)	0.018	0.44 (0.04, 0.85)	0.032
Age	0.01 (0.004,0.02)	0.007	0.01 (−0.0002, 0.019)	0.055
Sex	0.20 (−0.04, 0.43)	0.10	0.095 (−0.17, 0.36)	0.47
BMI	0.02 (−0.0001, 0.04)	0.050	0.019 (−0.002, 0.039)	0.071
GDR	−0.06 (−0.32, 0.20)	0.64	−0.11 (−0.37, 0.14)	0.39
Adiponectin	−0.07 (−0.19, 0.05)	0.22	−0.07 (−0.19, 0.05)	0.27
hsCRP	−0.87 (−3.62, 1.88)	0.53	−0.025 (−0.12, 0.07)	0.60

*Note*: Multivariate model includes tissue Na+, age, sex, BMI, log GDR, log adiponectin and log hsCRP.

Abbreviation: CI, confidence interval.

### 3.5 Effect of sex on the association of IMAT and tissue sodium content

Given that men had significantly higher IMAT, as well as, skin sodium content, a subgroup analysis was carried out to investigate a differential effect of sex. After stratification for sex, skin sodium was significantly associated with IMAT quantity in men, but not in women (Table 3). Muscle sodium was significantly associated with IMAT in both men and women, and there was no significant sex‐effect on this relationship.

**TABLE 3:** The associations between IMAT quantity and tissue sodium content stratified by sex.

	Women		Men		Sex interaction *p*‐value
	β (CI 95%)	*p*‐value	β (CI 95%)	*p*‐value	
Muscle sodium	0.85 (0.05, 1.65)	0.038*	1.32 (0.06, 2.58)	0.041*	0.51
Skin sodium	0.39 (−0.11, 0.88)	0.12	1.11 (0.55, 1.68)	0.000*	0.06

* Indicates two‐sided *p* < 0.05 meets significance criteria.

### 3.6 Effect of inflammatory markers and adipocytokines on the association of IMAT and tissue sodium content

**FIGURE 2:**
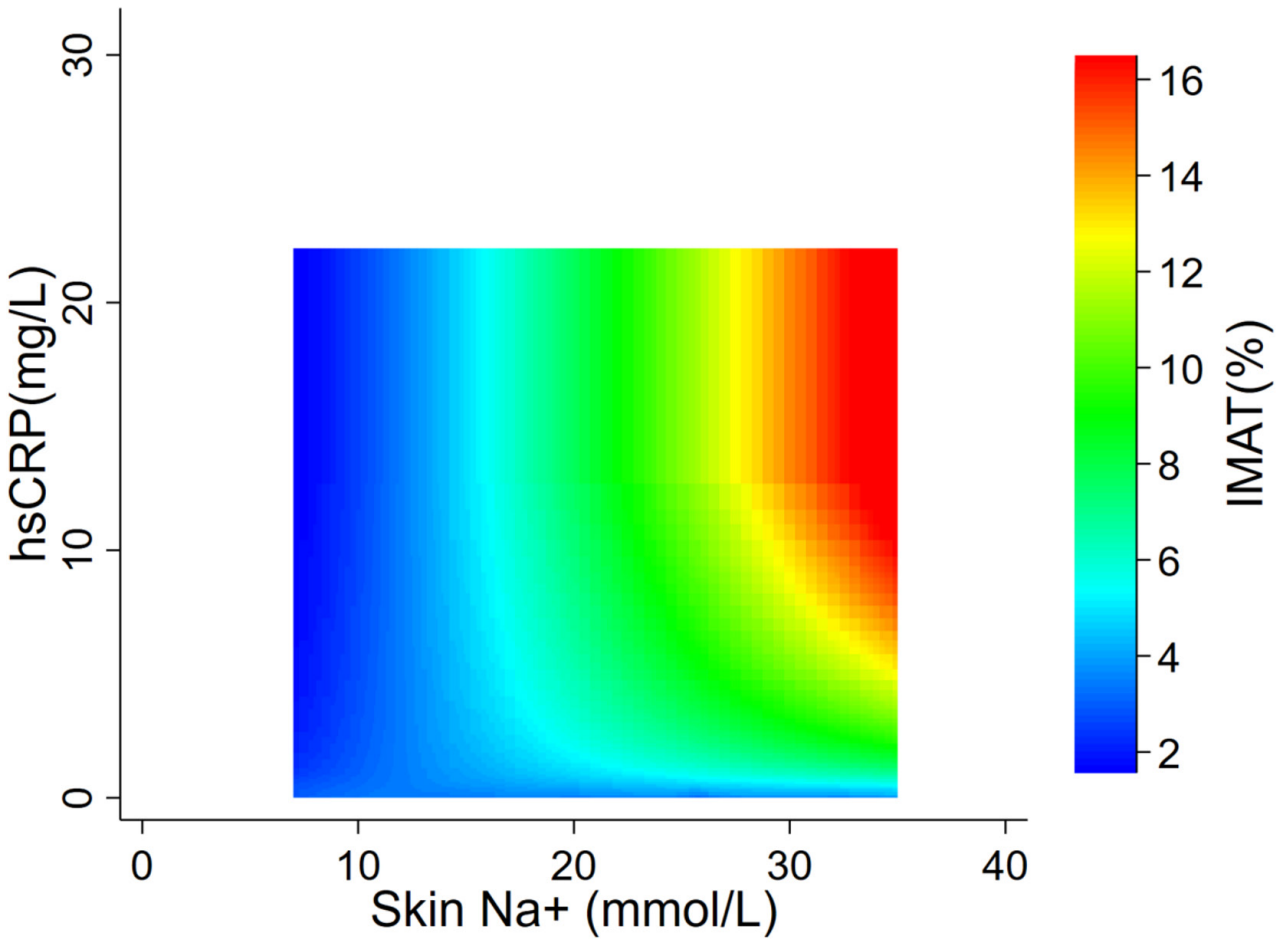
Interaction between skin sodium and plasma hsCRP for the association with IMAT. At higher hsCRP levels, there was a greater increase in IMAT per unit change in skin sodium. *p* for interaction 0.022. There was no significant interaction between muscle sodium and plasma hsCRP for the association with IMAT.

To test the hypothesis that inflammation may play a role in the relationship between skin and muscle sodium and IMAT accumulation, interaction analysis including serum inflammatory markers and adipocytokines was conducted. There was a significant effect modification of the association between skin sodium and IMAT by inflammatory markers hsCRP and IL‐6, such that the importance of skin sodium in determining IMAT quantity increased at high levels of serum inflammatory markers (*p* for interaction = 0.022 and 0.006 for hsCRP and IL‐6, respectively). A 10% increase in skin sodium content was associated with an increase in IMAT of 9.2% at a serum IL‐6 of 2.0 pg/mL, and of 14.1% at a serum IL‐6 of 4 pg/mL. Likewise, a 10% increase in skin sodium was associated with an increase in IMAT of 8.9% at a serum hsCRP level of 2 mg/L, and of 11.5% at a serum hsCRP level of 4 mg/L. Figure 2 depicts the change in the relationship between IMAT and skin sodium with changing levels of hsCRP. The interactions between skin sodium and inflammatory markers remained significant after adjustment for age, sex, and BMI. Inflammatory marker interaction was not observed in the models including muscle sodium. No interaction was found between the adipocytokines and skin or muscle sodium for the association with IMAT.

## 4 DISCUSSION

In this study, we investigated the association of lower‐extremity skin and muscle tissue sodium with IMAT in healthy individuals using a 3.0T multi‐nuclear MRI. Our data showed that tissue sodium content in both skin and muscle significantly associate with IMAT quantity independent of metabolic disease risk factors. An observation of the sex‐dependence of this relationship in our cohort revealed the association between skin sodium and IMAT was present only in men. We also observed that the association between skin sodium and IMAT was modified by circulating inflammatory markers IL‐6 and hsCRP. Higher serum levels of hsCRP and IL‐6 conferred a stronger association between skin sodium and IMAT. This effect modification was not observed between muscle sodium and IMAT.

The relationship between fat and sodium retention in the extremities of healthy individuals has important implications for potential underlying mechanisms. Exposure of adipocytes to high extracellular salt in vitro has been shown to stimulate adipogenesis and lipogenesis along with adipocytokine production and adipocyte inflammation (Lee et al., 2019). The high sodium microenvironment within the muscle may act as a driver of adipogenesis. We found that muscle sodium is strongly associated with IMAT; however, there was no association of muscle sodium with markers of whole‐body adiposity, including BMI, leptin, or adiponectin levels. Also, a study in mice found that high salt intake reduced abdominal fat accumulation (Cui et al., 2017). These findings suggest that the relationship between muscle sodium and IMAT may be mediated through local mechanisms. It is also possible that exaggerated sodium and fat storage co‐exist as markers of metabolic dysfunction in the muscle. Thus, in several chronic conditions such as aging, type 2 diabetes, and progressive kidney disease, there is increased tissue sodium as well as IMAT, suggesting overlapping mechanisms.

A growing body of evidence has shown an intricate cross‐talk between IMAT accumulation and inflammation within the muscle (Goodpaster et al., 2022). Among 2651 participants of the Health, Aging and Body Composition study, high thigh IMAT was associated with higher levels of IL‐6 and CRP (Beasley et al., 2009). Gene expression profiling and microRNA transcriptomes of various adipose tissue in pigs have shown IMAT deposition, like visceral adipose tissue, is associated with immune dysregulation and inflammation while subcutaneous adipose tissue deposition mainly affects carbohydrate and fatty acid metabolism (Li et al., 2012; Ma et al., 2013; Zhou et al., 2013). In a coculture system, human visceral adipose tissue induces skeletal muscle cell atrophy and IL‐6 and IL‐1β mediated inflammation (Pellegrinelli et al., 2015). Furthermore, IMAT may influence insulin resistance by increasing the secretion of inflammatory cytokines, such as plasminogen activator inhibitor‐1 and monocyte chemotactic protein‐1 (Sachs et al., 2019). A recent study also found that IMAT gene expression was associated with glucose homeostasis and insulin resistance (Lutter et al., 2023). The proinflammatory microenvironment induced by IMAT and other mediators may also trigger further IMAT accumulation.

An intriguing finding in our study is that inflammation may play a key role in the relationship between skin sodium and IMAT. Previous studies showed an association between excess tissue sodium in skin and muscle and circulating inflammatory markers in patients with advanced kidney disease (Sahinoz et al., 2020). Inflammatory markers are also associated with ectopic adipose tissue accumulation, including IMAT, in older adults (Beasley et al., 2009; Cartier et al., 2009; Koster et al., 2010). Recent in vitro and animal studies established that a high salt environment is a robust stimulus for immune cell activation (Ertuglu et al., 2022; Jantsch et al., 2015; Pitzer et al., 2022). In high extracellular sodium, equivalent to skin sodium concentrations found in mice fed with high sodium diet (Titze et al., 2004), sodium enters antigen‐presenting cells and triggers T cell activation, leading to the secretion of various proinflammatory cytokines, including IL‐1β and IL‐6, and an inflammatory state of both locally and systemically (Barbaro et al., 2017). The skin harbors a large pool of highly dynamic antigen‐presenting cells (Kashem et al., 2017) prone to salt‐induced activation, and high skin sodium content has been previously shown to facilitate inflammation and antimicrobial defense in animal studies (Jantsch et al., 2015). Inflammation is also known to drive adipose dysregulation (Zatterale et al., 2020). Thus, infiltration of immune cells into subcutaneous adipose tissue may prevent the storage of lipids by dysregulating pre‐adipocyte differentiation (Gustafson et al., 2009; Isakson et al., 2009), which may result in a spillover of lipids and accumulation in ectopic locations such as IMAT. It is conceivable that a low‐grade systemic inflammatory response caused by high‐skin sodium may accelerate or even promote the development of IMAT. We found that skin sodium is associated with IMAT only at higher levels of circulating inflammatory markers. Larger studies are required to validate these results and the exact nature of the interplay between skin sodium, inflammation, and IMAT.

We also found that the relationship between skin sodium content and IMAT quantity differed between males and females, and is only significant in males in our cohort. There are several possible explanations for such an observation. In our study, men were characterized by significantly higher skin sodium as well as IMAT, which is consistent with the published literature (Machann et al., 2005; Wang et al., 2017). It is well‐established that fat distribution is sex‐dependent; women display a gynoid fat distribution phenotype with higher subcutaneous fat deposition, while men predominantly have central adiposity (Hattori et al., 1991; Karastergiou et al., 2012). Most of our female participants were premenopausal with presumably high estrogen levels. Sex steroids have opposing effects on adipocytokine secretion (Law et al., 2014); leptin and adiponectin secretion are induced by estrogen and suppressed by androgens (Christen et al., 2018; Cnop et al., 2003). Although we also found significant differences in serum leptin and adiponectin levels among men and women, the associations of IMAT with skin and muscle sodium were independent of these variables. Sex steroids have differential effects on immunity as well (Straub, 2007). Estrogen is anti‐inflammatory and inhibits IL‐1β and IL‐6 secretion in monocytes (Pelekanou et al., 2016; Rogers & Eastell, 2001; Singh et al., 2022). If the link between skin sodium and IMAT is immune‐dependent, pre‐menopausal women could be partially protected from the high salt‐induced inflammatory response and related consequences. Other sex‐based differences that may be implicated in our findings include higher metabolism of free fatty acids and lipolysis in women (Wahrenberg et al., 1989) observed at both rest and exercise (Horton et al., 1998; Marinou et al., 2011; Nielsen et al., 2003). Nevertheless, our current findings are not sufficient to eliminate the possibility of a significant relationship between skin sodium and IMAT in healthy women due to their low level of skin sodium content and IMAT. Future studies are needed to demonstrate the relationship between skin sodium and IMAT in the postmenopausal population.

We have not found a correlation between dietary salt intake and tissue sodium storage in the skin or muscle. While high salt feeding has been shown to increase tissue sodium storage experimentally (Titze et al., 2004), such an association has not yet been confirmed in clinical studies. Whether and how dietary sodium intake relate with tissue sodium remains to be investigated in longitudinal clinical studies.

This study has several limitations. Our study population consisted of healthy individuals without metabolic, renal, or cardiovascular diseases that are known to be associated with high tissue sodium storage or IMAT, and observations may vary in disease states. Our analysis did not include an estimate of physical activity, which has been shown to influence IMAT (Chambers et al., 2020). Due to the cross‐sectional design, our findings offer very limited causal inference. Larger prospective studies with interventions for IMAT and sodium storage are needed to provide direct causal inferences. The strengths of our study include well‐powered sample size, availability of detailed metabolic parameters including inflammatory markers and adipocytokines, clinically‐standard metrics of insulin sensitivity, and the utilization of multi‐nuclear ^23^Na/1H MRI of tissue sodium and IMAT. The current results should therefore serve as a basis for future research into the pathways linking tissue sodium storage and IMAT deposition. These data could in turn motivate the study of preventive measures in individuals with cardiometabolic disease risk.

In conclusion, high tissue sodium in the muscle and skin of the lower extremities are associated with increased IMAT quantity in healthy individuals. The association between skin sodium and IMAT may be sex‐dependent and partially mediated by inflammation. Additional studies are needed to explore the underlying mechanisms of IMAT and tissue sodium deposition and to better understand its clinical relevance.

## AUTHOR CONTRIBUTIONS

TAI. and JLG. conceived and designed research; M.S., A.A., S.D. performed the investigation, L.E. analyzed the data, L.E., TAI. and JLG. interpreted the results, L.E. drafted the manuscript, M.S., A.A., S.M.D, A.G, M.D., C.C., E.A, M.P., R.C., M.M., A.K., D.H., F.L., J.T., TA.I and JLG. edited and revised the manuscript; all authors approved the final manuscript.

## FUNDING INFORMATION

This study was funded by AHA 14SFRN20770008, Vanderbilt O'Brien Kidney Center P30‐DK114809 and R01DK125794 from NIDDK, the Clinical Translational Science Award UL1‐TR000445 from the National Center for Advancing Translational Sciences, and the Veterans Administration Merit Award 5I01CX001755.

## CONFLICT OF INTEREST STATEMENT

TAI reports personal fees from Fresenius Kabi, Abbott Renal Care, and Nestle. JLG serves as a consultant for Pharvaris Gmbh.

## ETHICS STATEMENT

The study was reviewed and approved by the Vanderbilt University Human Subjects Institutional Review Board. All study procedures were conducted in accordance with the Declaration of Helsinki. All participants reviewed and signed informed consent prior to participation.

## Data Availability

This study is registered at https://clinicaltrials.gov/ct2/show/NCT02236520. The data will be made available from the corresponding author upon reasonable request.
